# Bumble Bee Fauna of Palouse Prairie: Survey of Native Bee Pollinators in a Fragmented Ecosystem

**DOI:** 10.1673/031.013.2601

**Published:** 2013-04-08

**Authors:** T. D. Hatten, C. Looney, J. P. Strange, N. A. Bosque-Pérez

**Affiliations:** 1 Invertebrate Ecology Inc., 121 W. Sweet Ave, Moscow, ID 83843; 2 Washington State Department of Agriculture, 1111 Washington St. SE, Olympia, WA 98504-2560; 3 USDA-ARS, Pollinating Insect Research Unit, 255 BNR, Utah State University, Logan, UT 84322-5310; 4 University of Idaho, Department of Plant, Soil, and Entomological Sciences, P.O. Box 442339, Moscow, ID 83844-2339

**Keywords:** Apidae, *Bombus*, diversity, grasslands, habitat fragmentation, Idaho, landscape context, pitfall traps, remnants, Washington

## Abstract

Bumble bees, *Bombus* Latreille (Hymenoptera: Apidae:), are dominant pollinators in the northern hemisphere, providing important pollination services for commercial crops and innumerable wild plants. Nationwide declines in several bumble bee species and habitat losses in multiple ecosystems have raised concerns about conservation of this important group. In many regions, such as the Palouse Prairie, relatively little is known about bumble bee communities, despite their critical ecosystem functions. Pitfall trap surveys for ground beetles in Palouse prairie remnants conducted in 2002–2003 contained considerable by-catch of bumble bees. The effects of landscape context, remnant features, year, and season on bumble bee community composition were examined. Additionally, bees captured in 2002–2003 were compared with historic records for the region to assess changes in the presence of individual species. Ten species of bumble bee were captured, representing the majority of the species historically known from the region. Few detectable differences in bumble bee abundances were found among remnants. Community composition differed appreciably, however, based on season, landscape context, and elevation, resulting in different bee assemblages between western, low-lying remnants and eastern, higherelevation remnants. The results suggest that conservation of the still species-rich bumble bee fauna should take into account variability among prairie remnants, and further work is required to adequately explain bumble bee habitat associations on the Palouse.

## Introduction

Arable grasslands have been disproportionately converted to intensive agriculture worldwide, and grassland ecosystems are critically threatened throughout the northern hemisphere (Noss et al. 1995; Tscharntke et al. 2002). Prairie habitat loss in North America has exceeded 95% in the tall-grass prairies of the central plains, and 63% in the mixedgrass prairies east of the Rocky Mountains (Samson and Knopf 1994; Robertson et al. 1997; Smith 1998). The lesser-known bunchgrass meadow steppe of the Palouse region in eastern Washington and northwestern Idaho, the Palouse Prairie, has been similarly impacted. Most of the prairie on the Palouse was converted to agriculture during the last century, taking advantage of the fertile soils and mild climate to develop a thriving farm economy. Estimates of native grassland loss on the Palouse range from 94% (Black et al. 1998) to greater than 99.9% (Tisdale 1961; Noss et al. 1995). Irrespective of the actual amount lost, remnant prairie, hereafter referred to as “remnants,” occurs as isolated and relatively small patches (0.25 hectares to 20 hectares is most typical) spread throughout the agricultural landscape (Looney and Eigenbrode 2012). Although conversion of grasslands to new farms has largely ceased, these remnants may face new threats from expanding nonagricultural development, ranging from exurban housing (Nielsen-Pincus et al. 2010) to wind-power turbines, continuous threats from invasive weeds (Lichthardt and Moseley 1997; Weddell and Lichthardt 1998), and more generally, the biological and social challenges typical of fragmented ecosystems (Donovan et al. 2009).

Despite their small size and isolation, Palouse prairie remnants support a diverse native flora of over 350 plant species (Lichthardt and Moseley 1997; Hanson et al. 2008), some of which are listed as globally imperiled or federally threatened (Lichthardt and Moseley 1997; Weddell and Lichthardt 1998). Though limited, studies indicate that rich invertebrate communities also persist in this resilient ecosystem (Hatten et al. 2006; Looney et al. 2009; Pocewicz et al. 2009; Sánchez de-León and Johnson-Maynard 2009; Looney and Eigenbrode 2011). Although the Palouse faunae remain poorly known, conserving invertebrates and their ecological functions and services is essential for sustaining the health of remnant habitats (Samways 2005). Insects have numerous functions in ecosystem processes, as part of natural predator/prey relationships, as decomposers or detritivores, and critically as pollinators.

Bees are the most ubiquitous and diverse insect pollinators, and bumble bees, *Bombus* Latreille (Hymenoptera: Apoidea), are the most species rich and abundant group of social bees native to temperate North America (Kearns and Thomson 2001). Bumble bees have structural and behavioral adaptations for pollen collection and transport, and forage on pollen to feed developing larvae (Michener 2007). Unlike many solitary bees, bumble bees forage throughout the season, pollinating a diverse flora. Native bees provide lucrative pollination services for production agriculture, potentially totaling over $3 billion per year in the USA alone (Losey and Vaughn 2006), and their pollination of non-cultivated plants is of inestimable value. Bees play a critical role in plant conservation, thus local or regional extinctions of bees can impact plant communities (Biesmeijer et al. 2006; Vamosi et al. 2006).

Despite the importance of bumble bees to native plant communities and agriculture, several North American species are in decline (Cane and Tepedino 2001; Colla and Packer 2008; Grixti et al. 2009; Cameron et al. 2011). Habitat loss and fragmentation contribute significantly to such declines, as do pesticide use and exposure to novel pathogens (Goulson et al. 2008; Cameron et al. 2011). Significant changes in bumble bee community composition and loss of genetic diversity have occurred in Illinois, as tall grass prairie was lost to agriculture (Grixti et al. 2009; Lozier and Cameron 2009). Bumble bee communities associated with small, isolated habitat remnants such as those found across the Palouse Prairie may be at similar risk, yet little is known about the bumble bee community in this habitat. Bee communities of remnant habitats are influenced by numerous factors, including the composition and quality of the surrounding landscape (i.e., the matrix) (Steffan-Dewenter and Tscharntke 1999; Steffan-Dewenter et al. 2002; Hines and Hendrix 2005; Hendrix et al. 2010). Bumble bees can travel up to 1.2 km (Knight et al. 2005) and routinely fly 450 m to 750 m between nest sites and floral patches (Walther-Hellwig and Frankl 2000). Hence, while floral diversity within habitats can be a strong predictor of bumble bee diversity, so too can density and diversity of floral resources in adjoining matrix habitats (Steffan-Dewenter et al. 2002; Hines and Hendrix 2005).

This study assessed the abundance, diversity, phenology, and distribution of bumble bees of remnant Palouse prairie, and compared species collected in this study with historical records from the region. Effects of landscape context, remnant features, year, and season on bumble bee community composition were examined. The recent data derive from pitfall traps employed in 2002–2003 to sample ground-dwelling invertebrates (Hatten et al. 2006) that also captured numerous bee species. Although not the focus of the initial study, the large number of bumble bees captured in these traps provides baseline data for future community-level studies on the Palouse, where virtually no work on pollinator communities has been previously conducted.

**Table 1.**
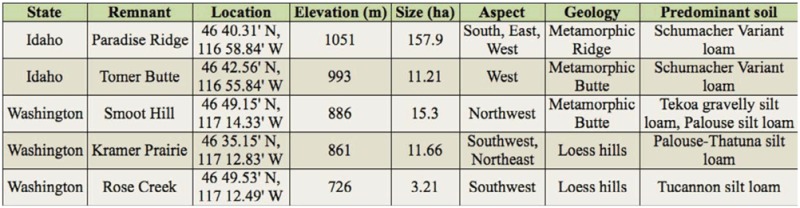
Characteristics of Palouse prairie remnants sampled in Latah County, ID and Whitman County, WA, 2002 and 2003.

## Methods and Materials

### Study sites

The Palouse bioregion extends over an 800,000-hectare region in eastern Washington and northwestern Idaho. It is characterized by hilly terrain of wind-blown loess overlaying 17-million-year-old basalt (Orr and Orr 2002). Buttes and hills comprised of more ancient metamorphic rock rise above the loess hills throughout the eastern Palouse, with soils that are typically thinner and rockier than the surrounding landscape (Donaldson 1980; Barker 1981; Orr and Orr 2002). The region receives over 430 mm of precipitation annually, and supports native meadow-steppe vegetation interspersed with patchy forest and shrub stands (Daubenmire 1970). Prairie remnants are numerous yet small, and are typically nestled on steep slopes surrounded by agriculture, or on the thin, unproductive soils of buttes and ridges (Looney and Eigenbrode 2012).

**Table 2.**
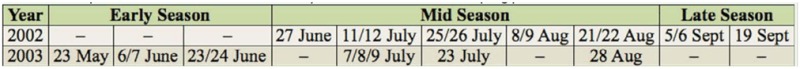
Sample collection dates, divided into early-, mid- and late-season sampling periods.

**Figure 1.**
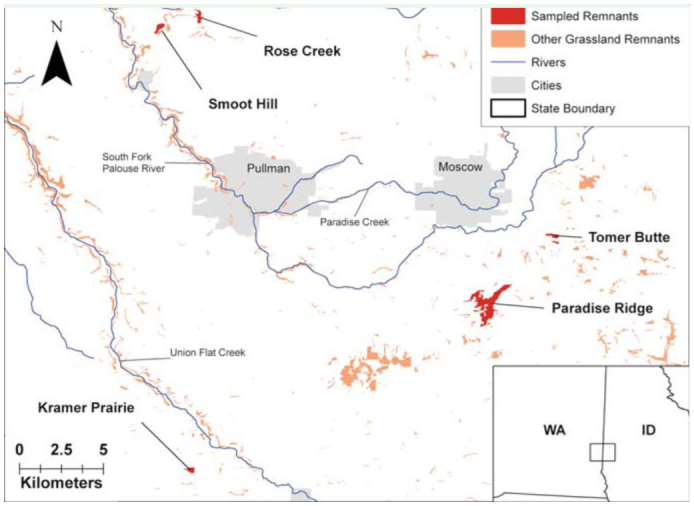
Collecting sites and other grassland/shrubland remnants in the southern part of the Palouse Prairie. High quality figures are available online.

Five prairie remnants were sampled during the summers of 2002 and 2003; Paradise Ridge and Tomer Butte in Latah County, Idaho, and Smoot Hill Preserve, Kramer Prairie Natural Area, and Rose Creek Preserve in Whitman County, Washington (Figure 1). These remnants differ in elevation by up to 300 m and range in size from 2.0 to 157 hectares (Table 1), but all contain a rich prairie flora with a strong bunchgrass component. The three highest remnants are found on buttes and ridges with relatively thin soils, while the two lowest are situated on loess hills (Donaldson 1980; Barker 1981).

### Sampling

Pitfall traps were installed along two transects within each remnant. Traps were constructed from 266 mL plastic cups with a white interior. Cup dimensions were 70 mm top diameter, 45 mm bottom diameter, and 95 mm depth. Traps were partially filled with propylene glycol and left open for seven days for a sampling period. There were seven one-week trapping periods in 2002 and six one-week trapping periods in 2003 (Table 2). There were minor differences in the configuration of trap transects between years. During 2002, each remnant had 18 traps arrayed as five trap pairs along one transect and four trap pairs along a second transect, while during 2003 each remnant had 16 traps arranged as four trap pairs on each of two transects. During both years, transects were positioned perpendicular to each other at Paradise Ridge, Tomer Butte, and Smoot Hill, and parallel to each other at Rose Creek and Kramer Prairie. Trap pairs were spaced every 50 m on a transect, with individual traps randomly located on either side of the transect.

Bumble bees were removed from traps and stored in ethanol until they were pin-mounted and labeled. The bees were identified to species, and the number of males, queens, and workers were determined at the USDA-ARS Pollinating Insect Research Unit in Logan, Utah. Voucher specimens are deposited in the USDA-ARS National Pollinating Insect Collection in Logan, Utah, Invertebrate Ecology's synoptic collection in Moscow, Idaho, the Washington State Department of Agriculture Collection in Olympia, Washington, and the W. F. Barr Insect Museum at the University of Idaho, Moscow, Idaho.

### Historical vs. current species records

The historical bee community was determined by searching the National Pollinating Insect Database (National Pollinating Insect Database 2011) for specimen records from Latah or Whitman Counties dated earlier than 2001. Only species with a minimum of 10 records in the database were used for the comparison, resulting in a total of 15 bumble bee species. In all, 2,408 historical specimen records were pooled to provide an indication of the bumble bee fauna that resided in the region prior to 2001. Current species composition was based only on bees collected during the 2002–2003 study period.

### Mean abundance comparison

Mean abundance was calculated as the average number of bees per trap per remnant. Since remnants were sampled on two unique dates during the early summer of 2003, multiple sampling dates during the midsummer of 2002 and 2003, and two unique dates during the late summer of 2002, data were organized into three distinct sampling periods: earlyseason, mid-season, and late-season (Table 2). Bumble bee abundance was compared between years using a mid-season-combined model, and compared within year using a dual-season-annual model. Both models employed a split-plot-in-time ANOVA (Proc GLM, SAS Institute 2001) to account for repeated sampling by remnant. Model terms in the mid-season-combined model were year, remnant and year*remnant, with the variable date (year* remnant) serving as the error term for Type III comparisons. In the dual-seasonannual model, model terms were season, date, season*date, with the variable date (season*remnant) serving as the error term for Type III comparisons. A Bonferroni adjustment was used for multiple comparison tests in both models (i.e., remnant vs. remnant within and between years or seasons) and reported *p*-values herein reflect adjusted statistics. Comparison of mean abundance by sampling date was not suitable due to insufficient degrees of freedom.

**Table 3.**
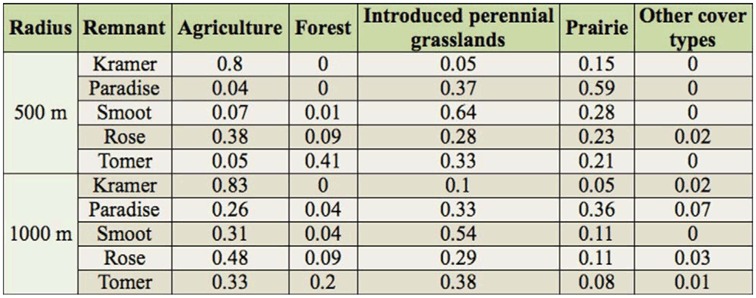
Proportion of different landscape covers within 500 m and 1000 m radii from sample remnants in the Palouse. “Other” cover types include houses, roads, rivers, etc.

### Diversity and ordination analysis

Rarefaction was used to standardize sample size by sampling season. For the 2002 data, rarefaction reduced sample size from 399 traps to 350 traps, or 70 traps per remnant in mid-season, and 177 traps to 165 traps, or 33 traps per remnant, in late-season. For 2003 data, rarefaction was not performed because sample size was equal among remnants and dates by season, except in Rose Creek during the mid-season. Sampling at Rose Creek was halted during the study due to competing research, and was not included in mid-season diversity analysis. The data were used to calculate species richness, community evenness, Shannon diversity index, and Simpson's index (Magurran 2004).

Rarefied count data by remnant and season were normalized by log transformation, combined into a dataset with the rarefied diversity data, and underlain with an environmental dataset containing nine landscape variables (Table 3, 6). Landscape data were derived from hand-digitizing aerial photographs for several land cover-types (e.g., agriculture, forest), and then extracting those data from nested 500 m and 1000 m diameter circles centered around each remnant (as in Steffan-Dewenter et al. 2002; see also Looney and Eigenbrode 2011). Soils data were taken from USDA soil surveys of Latah and Whitman Counties (Donaldson 1980; Barker 1981). Principal component analysis ordination was performed on the abundance/environmental data matrix using CANOCO 4.5 (ter Braak and Smilauer 2002). A species/diversity by remnant by landscape triplot was constructed for each sampling season in CANODRAW 4.5 (ter Braak and Smilauer 2002). Linear relationships among the four diversity metrics and the nine landscape variables within season were further assessed via correlation analysis (PROC CORR; SAS 2001).

## Results

A total of 1,192 bumble bee specimens representing 10 species were captured during the study. Fifteen species of *Bombus* have been recorded from Whitman and Latah counties; 67% of these were detected currently. The five missing species were *B. flavifrons* Cresson, *B. melanopygus* Nylander, *B. mixtus* Cresson, *B. occidentalis* Greene, and *B. vagans* Smith. Of the detected species, 751 individuals comprised of nine species were captured in 2002, and 441 individuals comprised of ten species were captured in 2003 (Table 4). *B. rufocinctus* Cresson was the most abundant species captured, accounting for approximately 76% of total catch in both years. The next most abundant species, *B. appositus* Cresson, comprised only 8% of the total catch in both years. In contrast, *B. californicus* Smith and *B. huntii* Greene were the least abundant species captured, both accounting for < 1% of trap catches during the study.

**Figure 2.**
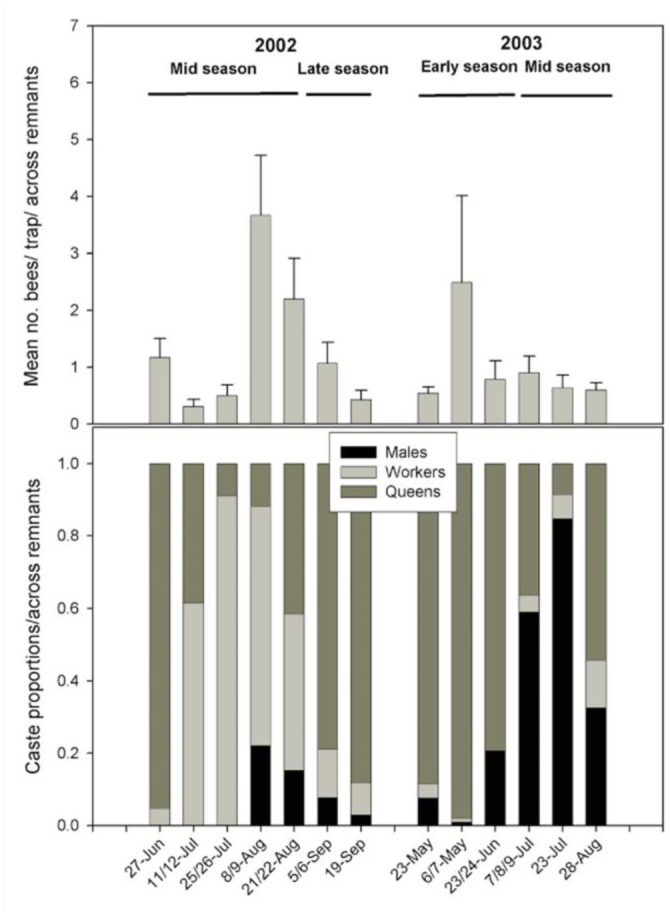
Mean number of bumble bees captured per trap and date across five prairie remnants during 2002 and 2003 (top), and relative proportions of the same bees by caste during this time period (bottom). Error bars ± SEM. High quality figures are available online.

Mean abundance by date could not be compared statistically, although densities clearly fluctuated by sample date. In 2002, capture rates peaked during the 8/9 August sample (Figure 2); only *B. bifarius* differed, with most captures made on the 21/22 August sample. In 2003, total *Bombus* catch rate peaked during the 7/8 June sample for most species. Exceptions were *B. nevadensis*, which was captured at a relatively constant yet low rate from early June and on, and *B. centralis*, which was most abundant in the 23/24 July sample. Seasonal dynamics were less apparent for rarer species (e.g., *B. huntii*) in both sample years.

**Table 4.**
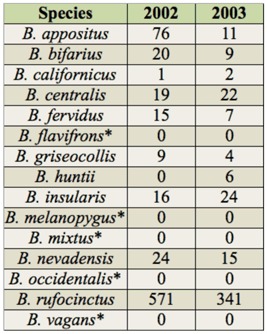
Total abundances of bumble bee species captured in 2002 and 2003 across prairie remnants and sampling seasons in Latah County, 1D and Whitman County, WA, and those species found historically in the region but not during the study as indicated by an asterisks and zeros in both data columns.

The relative proportions of bumble bee castes changed markedly among sample dates, seasons, and years (Figure 2). Queens were numerically dominant during early-season of 2003 and late-season of 2002. Workers were predominantly captured during the mid-season of 2002, but largely absent in 2003. Males were captured throughout 2003, accounting for approximately 20% of the total catch by the end of the early-season, and for 30–80% during the mid-season (Figure 2).

**Figure 3.**
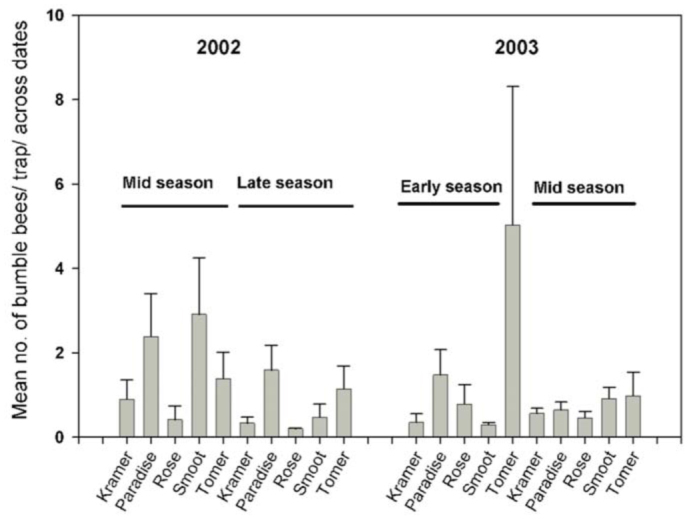
Mean number of bumble bees captured per trap across multiple sampling dates by prairie remnant during 2002 and 2003. Error bars ± SEM. High quality figures are available online.

ANOVA using the mid-season-combined model (Figure 3) revealed a significant year effect (*F*_19,36_ = 2.44, *p* = 0.04) on the bumble bees, (*t* = 0.02) with LS log mean densities higher in 2002 (0.8) than in 2003 (0.17). No significant remnant or year by remnant effects were observed. No significant effect of sampling season, remnant, or season by remnant interaction effect was identified with the dualseason-annual model during 2002. No sampling season or remnant effect was identified during 2003, but a significant season by remnant interaction effect was detected (*F*_4, 18_ = 3.05,*p* = 0.04). Nevertheless, means comparison tests revealed no significant differences in bee density by remnant in 2003, and only marginally significant differences between Tomer Butte and Kramer Prairie (*p* > 0.07) and Tomer Butte and Smoot Hill (*p* > 0.07) during the 2003 early-season. The same analysis for *B. rufocinctus* alone indicated no significant season or season by remnant effects, while the effect of remnant was significant (*F*_4, 18_ = 3.67, *p* = 0.02). *B. rufocinctus* mean abundance was greater at Tomer Butte than Kramer Prairie, Rose Creek, and Smoot Hill (*p* = 0.03; *p* = 0.05; *p* = 0.05, respectively). No other remnant pairs differed significantly.

Species richness by remnant ranged from four to eight species during the early- and midseasons of 2002 and 2003, and three to five species during the late-season of 2002 (Table 5). Species richness tended to be higher at Smoot Hill and Tomer Butte, while community evenness, Shannon diversity index, and Simpson's index were higher for Kramer Prairie and Rose Creek. These metrics were intermediate in value for Paradise Ridge.

**Figure 4.**
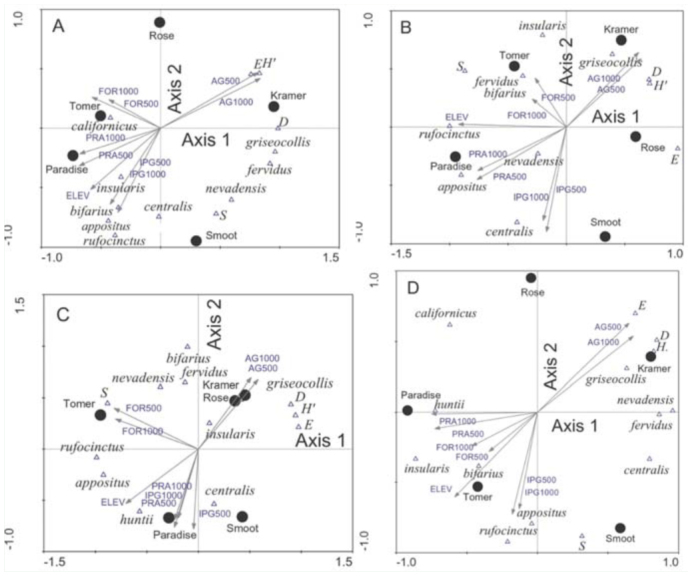
Species-prairie-landscape triplots based on principal component analysis of the bumble bee community found in Palouse prairie remnants of northwestern Idaho and eastern Washington during the mid-season (A) and late-season (B) of 2002, and the early-season (C) and mid-season (D) of 2003. S = species richness, E = community evenness, D = Simpson Index, and *H*′ = Shannon Index. High quality figures are available online.

Sample variances of the bumble bee community were adequately explained by principal component analysis. The combined variance explained by Axis 1 and Axis 2 was 37.7% and 74.5% (mid-season 2002), 98.8% and 99.6% (late-season 2002), 64.0% and 83.9% (early-season 2003), and 44.7% and 76.2% (mid-season 2003). Species composition gradients differed only slightly among seasons, especially for mid-season comparisons (Figure 4 A, D, respectively). Generally, *B. rufocinctus, B. appositus, B. bifarius, B. insularis*, and *B. californicus* were negatively correlated with Axis 1, as were Tomer Butte and Paradise Ridge. *B. griseocollis, B. fervidus*, and *B. nevadensis* were positively correlated with Axis 1, along with Kramer Prairie, Smoot Hill, and Rose Creek. In some cases, similarities in bee catch among remnants resulted in intermediate species scores. The Shannon diversity index, Simpson's index, and community evenness were positively correlated with Axis 1 and 2 in all plots, except late-season 2002 (Figure 4B), when community evenness was weakly negatively correlated with Axis 2. In contrast, the species richness was negatively correlated with Axis 1 and positively correlated with Axis 2 in lateseason 2002 and early-season 2003, and negatively correlated with Axis 2 and weakly positively correlated with Axis 1 in midseason of both years (Figure 4).

**Table 5.**
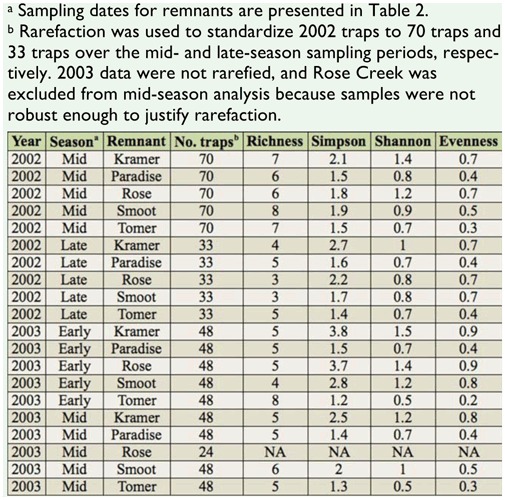
Biodiversity metrics for bumble bees captured in five prairie remnants during two sampling seasons, 2002 and 2003.

**Table 6.**
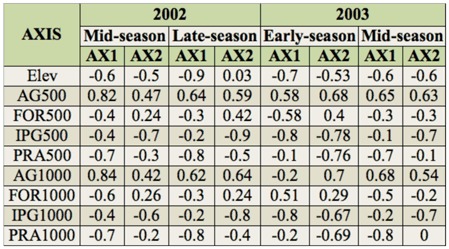
Pearson coefficients from regression of ordination axes against landscape variables that were centered around each remnant at 500 and 1000 m radii for sampling seasons within year.

Elevation, proportion of prairie at 500 m and 1000 m, proportion of forest at 500 m and 1000 m, and proportion of introduced grasslands at 500 m and 1000 m were each negatively correlated with Axis 1 in the species-remnant-landscape triplots (Table 6, Figure 4). The proportion of agriculture at 500 m and 1000 m was positively correlated with both axes. Variables measured at different radii were collinearly related; in principal component analysis, species gradients are not constrained by environmental variables (i.e., indirect gradient analysis), allowing examination of putative relationships without affecting the ordination (Lepš and Šmilauer 2003). Relative position and length of landscape vectors to prairie remnants reflects observed landscape context; e.g., higher elevation remnants are associated with higher proportions of forest, prairie, and semi-natural habitats, while lower elevation remnants are associated with agriculture-dominated landscapes.

Univariate correlation analysis (*N* = 5) showed significant positive correlations between Shannon diversity index and the proportion of agriculture at 500 m during midand late-season 2002 (*R* = 0.95, *p* ≤ 0.01; *R* = 0.93, *p* ≤ 0.01, respectively), and positive but nonsignificant correlations between community evenness and the proportion of agriculture at 500 m during mid-season of both years (*R* = 0.82, *p* < 0.1 *R* = 0.94, *p* < 0.1, respectively). Forest was significantly correlated with species richness during earlyseason 2003 at 500 m and 1000 *m* (*R* = *0.95*, *p* = 0.01; *R* = 0.88, *p* = 0.05, respectively).

## Discussion

### Historical and current community composition

The number of species (10) and abundance (1,192) of bumble bees captured during the study indicate that the Palouse prairie remnants continue to support a diverse bumble bee fauna. Ten of the 15 species historically recorded from Whitman and Latah Counties were detected in this study. The total species richness reported here is relatively high compared to similar studies in the western US and Midwest prairies. For example, research in the highly fragmented prairie ecosystems of Iowa found five bumble bee species in hill prairie remnants (Hendrix et al. 2010) and eight species in tall grass prairie remnants (Hines and Hendrix 2005). Similarly, a three-year survey of bee associates of flowering *Astragalus* and *Onobrychis* in eastern Washington located relatively close to the Palouse identified eight bumblebee species (Clement et al. 2006, but see Kimoto et al. 2012). Direct comparison of bumble bee fauna among studies is difficult, however, because sampling methodologies and effort differ markedly, and these factors affect all measures of biological diversity (Magurran 2004).

Of the five species that were historically present on the Palouse yet absent in this study, the lack of *B. occidentalis* is striking. Its absence mirrors recent surveys that found a broad geographic decline in the species across the western USA (Cameron et al. 2011). In fact, the most recent historical record for *B. occidentalis* on the Palouse is from 1977, indicating a long absence of this previously abundant bumble bee (National Pollinating Insect Database 2011). Four other species that were not detected during the study but are known from the Palouse bioregion include *B. flavifrons, B. melanopygus, B. mixtus*, and *B. vagans* (Table 4). These species were historically rare in the Palouse bioregion, so their absence from this study is not surprising. It is not clear from historical records if these species would even be expected in Palouse grassland habitats. *B. melanopygus, B. flavifrons*, and *B. mixtus* are more closely associated with the intermountain coniferous forests typical of eastern Latah County (Hobbs 1967; Strange, unpublished data). None of these four species have been recorded for some time on the Palouse, with the latest records for any of them occurring in 1954 (National Pollinating Insect Database 2011). In contrast, *B. vagans* is known to occur in grassland-forest interfaces (Hobbs 1967).

A species that was captured in surprisingly low numbers is *B. bifarius*, given its general abundance throughout the intermountain west (Cameron et al. 2011). In contrast, *B. rufocinctus* was more abundant in the samples collected than expected (Table 4). High relative abundances of *B. rufocinctus* is atypical of bumble bee assemblages in the western USA (Clement et al. 2006; Strange unpublished data), and its numerical dominance in this study is intriguing. This species is opportunistic in nest site selection, exploiting both subterranean environments such as abandoned rodent burrows, and epigeic habitats such as dry grass clumps (Macfarlane et al. 1994). This may be advantageous on the Palouse, particularly in habitats with coarse soils and limited rodent activity, and such nest-seeking behavior may explain its prominence in these collections.

Some of the differences in historical vs. observed species presence may also be a result of biases introduced by the study. These data include only a limited number of native prairie remnants and excluded human-altered landscapes, riparian corridors, and forested foothills, which may have biased the sample towards the collected species. Moreover, the historical records are based on many years of data compared with only two for the current study, and they derive from a broader suite of sampling methods than the pitfall method used in this study. Biases associated with pitfall trapping of epigeal fauna are widely acknowledged, yet as with most taxa (Spence and Niemela1994; Work et al. 2002), the capture efficiency of pitfall traps for bees is unknown. Pitfall traps are essentially similar to pan traps, with the latter method frequently used to sample bee communities. Pan traps typically use soapy water as a preservative and are installed at or above ground level. Pan color can have measurable effects on the bee fauna captured (Leong and Thorp 1999; Toler et al. 2005) and is known to result in taxonomic biases (Toler et al. 2005). Some studies have demonstrated that pan traps are less effective than netting when floral resources are abundant (Cane et al. 2000; Roulston et al. 2007; Wilson et al. 2008). The white trap color and propylene glycol preservative used in this study may have influenced capture rates in unknown ways. A limited net survey conducted during summer months subsequent to this study found slightly different dominance structure in the bee community; e.g., at Kramer Prairie during 2007, Strange (unpublished data) detected more queens of *B. nevadensis* and *B. fervidus* than of any other species, including *B. rufocinctus*, while at Smoot Hill during 2008 results were more comparable to the current study. Other authors have found that pan traps yield lower numbers of bumble bee species than sweep nets (see Wilson et al. 2008), and that large bees such as bumble bees can be poorly represented in such traps. Nevertheless, net surveys have limitations (collector bias among them) as well, making results difficult to compare short of rigorous pairwise trials.

### Abundance and caste composition

The lack of significant effect of sampling season on mean bumble bee abundance may be attributed to temporal variability of the catch data. The systematic sampling method used allowed a rigorous, regular sample over the course of the study. However, sampling on a calendar schedule increases sample variance caused by differences in weather and bee activity among dates, and perhaps by differences in sample size between seasons. Nevertheless, patterns typical of bumble bee phenology and colony cycles are apparent in the data. Bumble bees are eusocial bees that form colonies comprised of 20 to more than 300 individuals (Macfarlane et al. 1994; Kearns and Thomson 2001). Colonies are not maintained throughout the year, but are initiated each spring by overwintered gravid queens. Hence, populations tend to be low in the early-season when queens become active and establish nests (early June and before), high during mid-season when workers, males and new queens have emerged and are active (mid June-August), and again low during the late-season (late August-September). This general pattern is reflected in the data, although dominance of the catch by *B. rufocinctus* and inconsistent numbers of other species across the two sample years tended to obscure species-specific variability. Furthermore, a surge of *B. rufocinctus* queens—generally a late-spring species in other regions (Hobbs 1966; Colla and Dumesh 2010)—at one site during the 6– 8 May sample period are a testament to the importance of seasonal and site variability. It should be noted that since the earliest sample was collected on 23 May 2003, the data presented here underestimate abundance of species active in the early spring and limit the overall description of *Bombus* seasonality. Species that may have been more abundant and active in the early spring before sampling commenced include *B. bifarius, B. huntii*, and *B. griseocollis* (Hobbs 1966, 1967; Thorp et al. 1983; O'Donnell et al. 2000; Kearns and Thompson 2001).

Even with the limitations of the dataset, it is apparent that both bumble bee abundance and caste proportions differed appreciably between years. Approximately half as many bees were captured in the second year of the study than in the first, and the proportion of males captured in the second year was also much higher. Why abundance and caste proportions differed so strongly between years is unclear. A large number of bees including queens were captured during the study, raising the possibility that intensive sampling in 2002 affected populations in 2003. However, while intensive sampling could have reduced bee populations overall, it is not clear that this would have impacted caste proportions, particularly since numerous queens were trapped in both years (Figure 2).

One potential explanation for the preponderance of males in 2003 could be early transition to haploid egg production, presumably to increase mating success by producing numerous males (Bourke and Ratnieks 2001). Laboratory evidence indicates that extended diapause can stimulate this transition, although other factors, such as the emergence order of reproductives, play a significant role (Beekman and Van Stratum 2000; Duchateau et al. 2004). Data for other taxa (e.g., Carabidae) collected during this study indicate that a long, cold spring in 2003 depressed insect populations overall (Hatten et al. unpublished data) and likely extended diapause, possibly explaining both reduced bumble bee abundance and the high proportion of males in 2003.

### Site-specific differences and landscape-level patterns

Mean differences in bumble bee populations among remnants were generally not significant. The patchy distribution of bumble bees can inflate sample variances, and aggregating species counts across dates may have masked habitat effects. Furthermore, most species were too scarce in this study to detect strong differences among remnants. However, when examined independently of other species, mean abundance of the most abundant species (e.g., *B. rufocinctus*) was found to differ significantly among remnants, indicating that populations of some species are structured by remnant or landscape characteristics.

Patterns were more readily detectable at the community level than at the species level. Species richness was generally highest in eastern remnants, while peak diversity (Shannon diversity index or Simpson's index) was always highest in western remnants (especially Kramer Prairie and Rose Creek). Such patterns could be attributable in part to landscape context. Bee assemblages were correlated in principal component analysis with proportion of prairie, forest and/or introduced perennial grasslands in the matrix surrounding higher elevation remnants, and proportion of agriculture surrounding lower elevation remnants. Elevation was also correlated with species assemblage, although less consistently than land-cover types. Landcover variables and other important remnant characteristics (e.g., soil type and plant communities) are likely confounded. These variables were not tested explicitly in this study, primarily because detailed or appropriately scaled data are not available for all remnants. For example, data on floral diversity was not available for Rose Creek, and while soil types reported in Table 1 are derived from the most recent soil atlases, soil map units are at too coarse a scale to correlate with bumble bee community structure. The lower elevation remnants (Kramer Prairie and Rose Creek) are characterized by less gravelly soils, which certainly influence plant communities (Hanson et al. 2008) and perhaps ground-nesting rodents, which provide nests for many bumble bee species.

Correlations between landscape variables and bees must be considered cautiously, however, given the limited number of remnants sampled and inconsistent seasonal replication during the study. Nevertheless, observed patterns are suggestive of habitat selection by bumble bees on the Palouse. The forest-associated prairie remnants tend to be in landscapes with more frequent patches of natural habitat where conifer-shrub associations frequently intergrade with the bunchgrass-forb prairie remnants (Daubenmire 1970). This may increase effective patch size of prairie remnants by creating habitat and connectivity for bumble bees, supporting species otherwise more sensitive to habitat loss and thus higher species richness in these remnants. Bee species richness has been strongly correlated with both habitat size and connectivity (Steffan-Dewenter and Tscharntke 1999; Steffan-Dewenter 2003). Correlations observed at the 500 m radius were stronger than at 1000 m, which is consistent with general findings that landscape resources influence bumble bees and other bee populations most strongly at relatively local scales (Steffan-Dewenter et al. 2002; Hines and Hendrix 2005; Ricketts et al. 2008).

Lower elevation remnants sampled in the study were more isolated within the agricultural matrix, with a cropland dominated by wheat (both winter and spring wheat) and selffertilizing legume crops (especially spring peas during the study period). Bumble bee species can thrive in agroecosystems that offer flowering crops and nesting sites (Carvell et al. 2007; Broussard et al. 2011), and some open grassland species are adept at utilizing the small-scale resource elements that agricultural matrices provide (Diekötter et al. 2006). Local bumble bee communities could be sustained by the agricultural matrix, as evidenced by the higher diversity metrics seen in the lower-elevation remnants. However, this matrix likely provides only limited resources for bees given its prevalence of non-pollen or nectar producing crops, suggesting that bumble bee persistence is a function of the presence of weeds and native plants along roads and crop margins and/or resources available within the remnants. The later is most probable because floral diversity of grass/forb-dominated plant communities has been shown to be rich and to exceed that of shrub-dominated communities of Palouse Prairie (Hanson et al. 2008). Futhermore, diversity of flora is known to correlate with bumble bee diversity (Hendrix et al. 2010). Bumble bee community composition varied among remnants over the season. Initially, community composition of eastern remnants was influenced by higher abundance of *B. appositus, B. huntii, B. rufocinctus*, and *B. insularis*, while western remnants had higher catches of *B. griseocollis, B. nevadensis*, and *B. fervidus* (Figure 4C). This pattern weakened somewhat as relative abundance patterns shifted and more species associated with eastern remnants were detected in western remnants (Figure 4A, D). This shift was strongest for Smoot Hill during the midseason as the bumble bee community began to resemble more closely those from Tomer Butte and Paradise Ridge.

The observed shifts in mid-season community composition and species richness may reflect differences in geography and flora among remnants. Tomer Butte is west-facing, and appears to have locally accelerated floral phenology compared to the northwest facing Smoot Hill or the topographically diverse, higher elevation Paradise Ridge, perhaps providing conditions favorable for early season colonization and foraging. Smoot Hill is comprised of a mix of soil types (e.g., Tekoa gravelly silt loam and Palouse silt loam) and a topography conducive to seeps, forbs, and diverse woody species (e.g., rose, service berry, snowberry, and choke cherry), creating a unique microclimate and a prolonged bloom relative to Tomer Butte and potentially sustaining bumble bee activity over a longer time period. While bumble bees are able to warm themselves and fly at low temperatures (Heinrich 1975), nesting and foraging activities are nevertheless influenced by temperature and microclimate, and site-level edaphic variability may account for some of the observed dynamics.

One of the bees associated with agricultural land cover in this study, *B. fervidus*, is of interest because it is thought to be the primary pollinator of *Silene spaldingii* S. Watson, listed as threatened under the Endangered Species Act. This plant species has a limited distribution on the Palouse, with only small populations occurring on habitats with deep, grassland soils (U.S. Fish and Wildlife Service 2007). *S. spaldingii* is partially selfcompatible but also dependent upon pollinators, especially *B. fervidus*, for fruit development and seed set (Lesica 1993; Lesica and Heidel 1996). Among the remnants sampled, *S. spaldingii* is only known to occur in Kramer Prairie and Smoot Hill, both sites where the highest populations of *B. fervidus* were detected in this study, albeit in low numbers. While these data provide evidence that *B. fervidus* and *S. spaldingii* co-occur in prairie in the study area, additional studies would be required to elucidate plant-pollinator relationships.

## Conclusions

Bumble bee conservation has become a significant concern in Europe and North America, particularly in fragmented ecosystems such as the Palouse. Bumble bee conservation planning in many ecosystems is limited by poor understanding of species composition, faunal changes over time and how communities persist in fragmented ecosystems, requiring insights from community, landscape, and molecular ecology. The results presented here are drawn from a sample of five prairie remnants and an atypical bee sampling method. Nevertheless, these data suggest that Palouse prairie remnants continue to harbor diverse bumble bee communities, and offer insights that can inform further study and conservation planning on the Palouse. Perhaps chief among these is that potential differences in bumble bee assemblages among remnants appear to be linked to landscape context and site-level characteristics, and such variability should be explicitly examined in further studies and considered when conducting conservation planning.
